# Reply to Crespo, J.; Iruzubieta, P. Comment on “Orozco-Beltrán et al. Evaluation, Management and Therapeutic Approach of Cardiovascular–Kidney–Metabolic Syndrome: A Multidisciplinary Delphi Expert Consensus. *J. Clin. Med.* 2025, *14*, 8930”

**DOI:** 10.3390/jcm15197421

**Published:** 2026-09-24

**Authors:** Domingo Orozco-Beltrán, Borja Quiroga, Alberto Esteban-Fernández, Ana Lorenzo Almorós, Virginia Bellido, Teresa Benedito Pérez de Inestrosa, Rubén de Haro, Xoana Taboada, Juan Carlos Romero-Vigara

**Affiliations:** 1 Department of Clinical Medicine, School of Medicine, Miguel Hernández University, San Juan de Alicante, 03550 Elche, Spain; 2 Service of Nephrology, University Hospital of La Princesa, 28006 Madrid, Spain; borjaqg@gmail.com; 3 RICORS2040 (Belonging to the RICORS [Redes de Investigación Cooperativa Orientada a Resultados en Salud] Program), Instituto de Salud Carlos III, 28029 Madrid, Spain; 4 Service of Cardiology, Severo Ochoa University Hospital, 28914 Leganés, Spain; athalbertus@gmail.com; 5 Facultad de Ciencias de la Salud, Universidad Alfonso X El Sabio, 28691 Villanueva de la Cañada, Spain; 6 Service of Internal Medicine, Gregorio Marañón General University Hospital, Complutense University of Madrid, 28007 Madrid, Spain; alorenzoa@salud.madrid.org; 7 Clinical Management Unit of Endocrinology and Nutrition, Institute of Biomedicine of Sevilla (IBiS), Virgen del Rocío University Hospital, CSIC, University of Sevilla, 41013 Sevilla, Spain; virginiabellido@gmail.com; 8 Médico de Familia, Servicio Andaluz de Salud, 18013 Granada, Spain; teresa.benedito@gmail.com; 9 Medical Affairs Department, Boehringer Ingelheim, 08174 Sant Cugat del Vallés, Spain; ruben.de_haro@boehringer-ingelheim.com; 10 Medical Department, Eli Lilly and Company, 28108 Alcobendas, Spain; taboada_xoana@lilly.com; 11 Health Service of Aragon, 50017 Zaragoza, Spain; jcromerovigara@hotmail.es; 12 IISA—Aragon Health Research Institute, 50009 Zaragoza, Spain

We would like to thank Crespo and Iruzubieta for their thoughtful and constructive comment [1] on our recently published multidisciplinary Delphi consensus on cardiovascular–kidney–metabolic (CKM) syndrome [2]. Their timely contribution highlights the growing relevance of liver health within the integrated cardiometabolic framework and provides valuable insights for future refinement of CKM management.

First, we would like to emphasize that our consensus was conceived as a pragmatic and clinically oriented initiative aimed at translating the emerging CKM construct into actionable recommendations within the Spanish National Health System. The Delphi methodology allowed us to gather the perspectives of 70 experts in five key specialties, achieving consensus in 89% of statements, thereby ensuring both robustness and applicability in real-world clinical practice. To date, these are the only existing comprehensive recommendations worldwide regarding CKM syndrome.

We fully agree with the authors on the increasing importance of the hepatic axis in CKM syndrome. Indeed, our panel explicitly recognized metabolic dysfunction-associated steatotic liver disease (MASLD) as a relevant component and highlighted its integration as a future action area. As correctly pointed out, the evolving evidence base—including recent international consensus and policy developments [3,4,5], together with recommendations and consensus documents from the three Primary Care Societies in Spain [6,7,8]—supports a more definite incorporation of liver disease into the CKM framework.

This interpretation should be placed within the conceptual framework available when the Delphi process was conducted. The panel composition and the structure of the questionnaire were aligned with the original American Heart Association (AHA) definition of CKM syndrome [9], which did not explicitly include the liver as a core component. Consequently, the consensus reflects the boundaries of the CKM concept at that time while providing a basis that can accommodate subsequent evidence rather than a definitive model.

Importantly, our work already incorporates relevant hepatic assessment elements, such as the inclusion of FIB-4 in the basic analytical profile, which achieved a high level of agreement among experts. This decision reflected the recognition of the prognostic relevance of liver fibrosis within the CKM spectrum and provided a practical entry point for further operational development [10].

We acknowledge the authors’ proposal to operationalize liver-related parameters—particularly FIB-4—through structured diagnostic and referral pathways. A future revision could, for example, incorporate a stepwise algorithm in which an indeterminate or elevated FIB-4 prompts second-line assessment with transient elastography or the Enhanced Liver Fibrosis test, followed by referral to Hepatology when the probability of advanced fibrosis is high. We consider this a valuable and feasible extension of our consensus, especially within integrated care models supported by electronic health records and linked to predefined multidisciplinary referral criteria, building on the digital infrastructure strongly endorsed by our panel.

At the same time, we would like to underline that one of the main strengths of our Delphi consensus is its balance between scientific rigor and feasibility. By prioritizing broadly accepted, implementable measures across different specialties, the document aims to facilitate adoption in routine clinical practice. In this context, incremental integration of emerging components, such as the hepatic axis, may enhance uptake and sustainability.

It is important to contextualize our findings within the conceptual framework available at the time of the Delphi process and, at the same time, consider the rapid evolution of scientific knowledge over the last few months. In line with this evolution, since completion of our consensus, the 2026 guidelines on prevention, detection, evaluation, and management of CKM syndrome led by the American Heart Association (AHA), American College of Cardiology (ACC), American Diabetes Association (ADA), and American Society of Nephrology (ASN) have been published [11]. They specifically recognize MASLD, together with conditions such as pre-heart failure and obstructive sleep apnea (as well as other conditions, such as pre-HF and OSA), as part of the CKM syndrome and identify FIB-4 as a relevant tool. Moreover, they also highlight earlier identification of chronic kidney disease (CKD) and MASLD as an area requiring further improvement, consistent with the priorities identified in our consensus.

We therefore interpret the comment by the authors as a constructive contribution that reflects the natural and welcome evolution of the field. Their proposals are fully aligned with the spirit of our work, offer a practical roadmap for updating the hepatic dimension of the consensus without altering its original purpose or pragmatic orientation, and emphasize the crucial importance of addressing cardiovascular risk. As stated in our manuscript, periodic revisions of the consensus will be necessary to incorporate new evidence and expand the CKM framework toward a more comprehensive, truly multiorgan model.

In conclusion, our Delphi consensus represents a foundational step toward integrated CKM care. The incorporation of the hepatic dimension and an actionable assessment pathway in future revisions proposed by Crespo and Iruzubieta reinforces the need for continued multidisciplinary collaboration and supports the progressive refinement of this framework.

## Data Availability

No new data were created or analyzed in this study. Data sharing is not applicable to this article.

## References

[1] Crespo J., Iruzubieta P. (2026). Comment on Orozco-Beltrán et al. Evaluation, Management and Therapeutic Approach of Cardiovascular–Kidney–Metabolic Syndrome: A Multidisciplinary Delphi Expert Consensus. *J. Clin. Med.* 2025, *14*, 8930. J. Clin. Med..

[2] Orozco-Beltrán D., Quiroga B., Esteban-Fernández A., Lorenzo Almorós A., Bellido V., Benedito Pérez de Inestrosa T., de Haro R., Taboada X., Romero-Vigara J.C. (2025). Evaluation, management and therapeutic approach of cardiovascular–kidney–metabolic syndrome: A multidisciplinary Delphi expert consensus. J. Clin. Med..

[3] Zhou X.-D., Fan Q.-Y., Targher G., Byrne C.D., Chen Q.-F., Shapiro M.D., Dong Y., Sung K.-C., Lip G.Y.H., Lazarus J.V. (2026). MASLD as a systemic metabolic disease: Expanding the scope of cardiovascular-kidney-metabolic (CKM) syndrome. Sci. China Life Sci..

[4] Theodorakis N., Nikolaou M. (2025). From Cardiovascular-Kidney-Metabolic Syndrome to Cardiovascular-Renal-Hepatic-Metabolic Syndrome: Proposing an Expanded Framework. Biomolecules.

[5] Arragan Lezama C.A., Jaramillo Ramos J.J., Armas Eguizábal D.A., Ovando X.S.S., Villagrán J.C.M., Caiza C.J.M. (2025). Cardiovascular-Renal-Hepatic-Metabolic Syndrome: Interlinked Pathophysiology and Integrated Management Approach. Cureus.

[6] Paciente Cardiorrenal Metabólico. Recomendaciones SEMERGEN. AAFF_Recomendaciones SEMERGEN_Cardiorrenal CORR_80+cub.indd. https://semergen.es/files/docs/biblioteca/guias/2024/actualizacionRecomendacionesSemergen.pdf.

[7] Consenso Sobre el Manejo del Síndrome Cardio, Renal, Hepático y Metabólico en Atención Primaria. SEFAP, CAMFiC y AIFIC. ITEM_22600.pdf. https://gestor.camfic.cat/uploads/ITEM_22600.pdf.

[8] Documento SEMG. Ecografía Clínica en el Síndrome Cardio-Renal-Hepato-Metabólico (CRHM) para Médicos de Familia. Ecografía Clínica en el Síndrome Cardio-Renal-Hepato-Metabólico (SCRHM) para Médicos de Familia. https://www.semg.es/images/2026/documentos/Eco_clnica_SCRHM_SEMG.pdf.

[9] Ndumele C.E., Rangaswami J., Chow S.L., Neeland I.J., Tuttle K.R., Khan S.S., Coresh J., Mathew R.O., Baker-Smith C.M., Carnethon M.R. (2023). Cardiovascular–kidney–metabolic health: A presidential advisory from the American Heart Association. Circulation.

[10] Cao Z., Chen B., Zhou J., Jin J., Abramov D., Chen S., Huang P., Zhou J. (2026). Liver fibrosis biomarkers as a prognostic tool beyond the defining components in cardiovascular-kidney-metabolic syndrome. Am. J. Prev. Cardiol..

[11] Ndumele C.E., Rodriguez F., Dixon D.L., Khan S.S., Mukherjee D., Bajaj M., Bangalore S., Bozkurt B., Breathett K., Clarke S.L. (2026). 2026 AHA/ACC/ADA/ASN Guideline for the Prevention, Detection, Evaluation, and Management of Cardiovascular-Kidney-Metabolic Syndrome: A Report of the American College of Cardiology/American Heart Association Joint Committee on Clinical Practice Guidelines. J. Am. Coll. Cardiol..

